# Does change in heart rate and blood pressure during adenosine stress perfusion cardiovascular magnetic resonance (A-CMRP) imaging predict perfusion defects?

**DOI:** 10.1186/1532-429X-14-S1-M5

**Published:** 2012-02-01

**Authors:** Krishnaraj S Rathod, Ceri Davies, Steffen E Petersen, Mark Westwood, Neha Sekhri

**Affiliations:** 1 Cardiology, London Chest Hospital, London, UK

## Summary

Peripheral haemodynamic response during adenosine stress CMR perfusion does not vary among those with or without perfusion defects identified during routine clinical practice.

## Background

Haemodynamic response to adenosine infusion during CMR stress perfusion continues to be used as surrogate to determine adequacy of hyperaemia in clinical setting. Our aim was to determine the proportion of patients who achieve pre-specified adequate response to adenosine (increase in heart rate (HR) by ≥ 10 beats or a drop in systolic blood pressure (SBP) by 10mm Hg from baseline reading) and if this varies in patients identified with and without perfusion defect in a clinical setting.

## Methods

We prospectively identified 94 consecutive patients undergoing A-CMRP, 7 of who were excluded because of missing data. 87 patients underwent first pass CMRP on a Philips Achieva CV 1.5 T MR scanner (Philips, The Netherlands), with standardised SCMR acquisition protocol infusing adenosine at 140 μg/kg/min for 3 minutes with HR and SBP recorded at baseline, one, two and three minutes. All patients confirmed abstinence from caffeine and chocolate for >24 hours and no patients were on theophylline or dipyridamole. Descriptive statistics and logistic regression were performed.

## Results

67 (77%) patients achieved the pre-specified adequate haemodynamic response and this was mainly driven by increase in heart rate.

The OR (adjusted for age) for predicting perfusion defect by adequate haemodynamic response in patients with and without CAD was 2.5 (0.58, 10.40, p=0.21) and 3.26 (0.48, 22.13, p 0.20) respectively.

## Conclusions

Our study shows that in patients undergoing adenosine (140 μg/kg/min) stress perfusion, there is no significant difference in peripheral haemodynamics in patients identified with or without perfusion defect and challenges their use as a marker of hyperaemic response. It remains to be seen whether high dose adenosine will influence these findings and change clinical practice.

## Funding

No funding was obtained for this study.

**Table 1:** 

Total cohort N=87	Adequate response N=67 (77%)	Inadequate response N=20 (23%)	P value
Age (median)	60 (IQR 47, 72)	65 (IQR 50, 72)	0.65
Known CAD	34 (51%)	10 (50%)	0.93
Perfusion defect present	32 (48%)	6 (30%)	0.16
Good LV function (>55%)	58 (89%)	12 (60%)	0.01

**Table 2:** 

In those with known CAD N=44	Perfusion defect present N= 25(57%)	No perfusion defect N= 19(43%)	P value
Age (median)	65(IQR 50-72)	69(IQR 51-75)	0.69
Male	22(88%)	17(90%)	0.88
Rest SBP - mean SBP over 3 minutes of adenosine	-5.0 (IQR -9.0, 1.0)	-4.0 (IQR -8.0, 4)	0.16
Rest HR - mean HR over 3 minutes (beats per minute)	13 (IQR 10, 20)	14 (IQR 7, 24)	0.93
Patients with no Known CAD N= 43	Perfusion defect present N= 13 (30%)	No perfusion defect N=30 (70%)	
Age (median)	69 (IQR 56, 73)	54 (IQR 43, 67)	0.03
Male	8(62%)	14(47%)	0.37
Rest SBP - mean SBP over 3 minutes of adenosine	-10.3 (IQR -16.67, 0)	0 (IQR -7.0, 8.5)	0.23
Rest HR - mean HR over 3 minutes (beats per minute)	16 (IQR 13, 23)	21 (10, 27)	0.46

